# The Role of Mucins in Cancer and Cancer Progression: A Comprehensive Review

**DOI:** 10.3390/cimb47060406

**Published:** 2025-05-29

**Authors:** Clare Chen, Ameena Patel, Lusine Demirkhanyan, Christopher S. Gondi

**Affiliations:** 1 Department of Internal Medicine, University of Illinois College of Medicine Peoria, Peoria, IL 61605, USA; cchen448@uic.edu (C.C.); apate426@uic.edu (A.P.); lusinhd@uic.edu (L.D.); 2 Department of Surgery, University of Illinois College of Medicine Peoria, Peoria, IL 61605, USA; 3 Departments of Health Science Education and Pathology, University of Illinois College of Medicine Peoria, Peoria, IL 61605, USA; 4 Health Care Engineering Systems Center, The Grainger College of Engineering, University of Illinois at Urbana-Champaign, Urbana, IL 61801, USA

**Keywords:** mucin, O-glycosylation, metastatic cascade, antibody therapeutics

## Abstract

Mucin, a heavily glycosylated glycoprotein, serves an important function in forming protective and immune defense barriers against the exterior environment on epithelial surfaces. While secreted-type mucins are involved in mucous production, transmembrane mucins, which contain O-glycosylated tandem repeats, play a pivotal role in cellular signaling, especially in immune modulation and mediating inflammatory response. However, dysregulation in mucin expressions, such as MUC1, MUC2, MUC4, MUC5AC, and MUC16, have been observed in many cancer cells. More specifically, alterations in the expression and glycosylation of MUC1 have been associated with the upregulation of pathways involving the cell proliferation, angiogenesis, migration, and invasion of cancer cells. With mucin’s extensive involvement in cancer biology, several mucin biomarkers, such as CA125, CA19-9, and CEA, have been utilized as diagnostic and prognostic monitoring biomarkers in ovarian, pancreatic, and colon cancer. Vaccines and antibody therapy against abnormal mucin glycosylation have also been investigated for potential therapy for mucin-related cancers that are resistant to traditional chemotherapy agents. Despite the lack of specificity in mucin biomarkers and challenges in efficient drug delivery systems, the current advancement in mucin-targeted immunotherapy highlighted the pivotal potential in developing therapeutic targets to improve cancer prognosis.

## 1. Introduction to Mucins and Cancer

Mucus, secreted by goblet cells, is a viscous secretion that covers epithelial surfaces throughout the body. Its main components are water, salts, immunoglobulins, proteins, and mucins [1,2]. Mucin’s high molecular weight and heavily O-linked glycoproteins provide stable structure and protective function to mucosal surfaces [2]. As a primary component of mucus, mucins form a gel-like apical coat that lines the interface between the epithelium and the external environment [3]. They contribute to the viscoelasticity and hydration of the mucus barrier, acting as bio-adhesives and molecular sieves.

Over the past five decades, studies have demonstrated the dysregulation of mucins in the progression of human malignancies, highlighting their roles in inflammation, cancer recognition, and therapy resistance [4]. Elevated mucin levels are observed in the mucus-secreting cells of gastric, colorectal, and pancreatic adenocarcinomas, affecting the progression of cancer cells in these tissues [5,6,7].

For instance, MUC1 binds to E-cadherin and alters its function, acting as a sequestration factor for β-catenin, a key protein in the Wnt signaling pathway. The 20 μm mucin-rich pericellular halo around cancer cells provides a stealth cloak against the immune system and a slippery barrier to drug penetration [2,8]. Within the cancer microenvironment, tumor cells are encapsulated in mucus [3]. Cleared mucus, including recombinant mucins, can elicit varying immune responses, from strong activation to negative regulation [9,10]. Consequently, clearing infiltrated mucin families is exploited in various pathophysiological conditions [4,11,12].

### 1.1. Definition and Classification of Mucins

Mucins are highly glycosylated proteins commonly associated with the apical cell surface [9,10]. These gel-forming glycoproteins protect epithelial surfaces from solute particles, chemical irritants, pathogens, and mechanical stress [10]. Mucins are broadly classified into two main categories: secreted and transmembrane. Secreted mucins form a physical protective barrier with a viscous gel at the epithelium, most commonly in the gastrointestinal and urinary systems. Transmembrane mucins contribute to this protective gel through their O-glycosylated tandem repeats that extend above the apical cell surface [4]. Secreted mucins, such as MUC2, MUC5AC, MUC5B, and MUC6, are encoded by a cluster of genes on chromosome 11q15 [11]. Due to their shared similarity in dimerization, domain organization, pH-dependent assembly, and protective function, secreted mucins are proposed to share a common ancestor with the von Willebrand factor (vWF) [12]. Secreted mucins can be divided into monomeric and gel-forming mucins [13]. Monomeric mucins, such as MUC7, found in saliva [14], and MUC20, present in the kidney and urinary tract [15], serve specialized functions in their respective locations. Gel-forming mucins, including MUC2, MUC5AC, and MUC5B, are primarily involved in mucus production in the respiratory and gastrointestinal tracts [16]. These mucins adhere to the cell surface to protect the epithelium from pathogenic and mechanical injuries [17,18]. Their gel-like properties enable them to trap pathogens and particulate matter, facilitating their removal from the body.

Transmembrane mucins, such as MUC1, MUC13, and MUC16, are hydrophobic and attached to the cell surface [19]. The highly glycosylated extracellular and intracellular domains also enable them to participate in signaling pathways [20]. MUC1, for example, interacts with various intracellular signaling molecules, influencing cellular responses to external stimuli and playing a role in cell adhesion and immune response modulation [20,21]. This multifunctionality makes transmembrane mucins critical in maintaining epithelial integrity and responding to environmental changes.

### 1.2. Overview of Cancer and Cancer Progression

Epithelial cells line the surface of the epithelium, playing essential roles in protection, cell signaling, inflammation, and infection [22]. These cells are single-layered, exhibit apical-basal polarity, and have high turnover rates [23,24]. Under normal conditions, mucins are located at the epithelium apical surface, facing the external environment, while receptor tyrosine kinases (RTKs) are situated on the basolateral side. Chronic stress and inflammation can cause epithelial cells to lose their polarity, leading to the repositioning and interaction of apical mucins with basolateral proteins [25]. This interaction between mucins and RTKs promotes the survival of inflamed tissue and causes goblet cell hyperplasia, resulting in the hypersecretion of mucins [26]. The loss of polarity, along with the disruption of tight junctions and adherens junctions, triggers an epithelial–mesenchymal transition (EMT). EMT endows cells with characteristics of cancer cells, further activating cellular proliferation, differentiation, and survival mechanisms [25,26,27,28,29,30,31]. Recent research has extensively studied the role of mucin glycoproteins (MUC) in cancer. For example, the activation of ERBB2 RTK by transmembrane MUC1 following the loss of cellular polarity has been linked to the disruption of tight and adherens junctions mediated by E-cadherin [25,31,32]. These alterations in mucin expression and interactions contribute significantly to cancer formation and progression. The transformation from normal to neoplasia occurs incrementally, advancing stepwise through stages of hyperproliferation, dysplasia, loss of differentiation, transformation, and metastasis [33,34]. These changes lead to the development of various subtypes of colonic tumors, including hyperplastic/serrated adenomas, tubular adenomas, tubulovillous adenomas, villous adenomas, and adenocarcinomas [35]. Understanding the mechanisms underlying these transformations, particularly the roles of mucins and epithelial cell polarity, provides valuable insights into the development and progression of cancer, which is crucial for developing targeted therapies and diagnostic tools to combat cancer effectively.

## 2. Structure and Function of Mucins

Mucins are structurally characterized as large, viscous glycoproteins primarily composed of carbohydrates and amino acids linked via O-glycosylation [36]. At the molecular level, mucins are elongated, rod-shaped molecules with a linear polypeptide core [37]. The glycosylated regions, which often contain simple disaccharides like sialic acid, maintain the mucin molecule’s configuration, bind to water, promote hydration, and contribute to gel formation [36,37]. Proline residues play a crucial role in ensuring proper unfolding during the process of O-glycosylation. In contrast, non-glycosylated regions are rich in cysteine, which is vital for polymerization [3]. This polymerization is critical for the aggregation of intestinal [38] and tracheobronchial mucins [39].

Both intramolecular and intermolecular interactions are essential for mucin’s structural organization, including the cross-linking of mucin polymers leading to gel formation. Mucin secretions possess significant protective and lubricative properties. In the upper gastrointestinal (GI) tract, such as the stomach, mucins protect epithelial cells from the corrosive effects of digestive acids [40]. In the lower GI tract, particularly in the colon, mucins form a protective interface between host cells and the assemblage of gut microbiota and bacterial antigens [41,42]. The extensive mucin glycosylation serves in host defense by maintaining gut homeostasis and preventing pathogen invasion [13]. More specifically, the carbohydrate chains on mucins act as decoy receptors for pathogens, preventing their adherence to epithelial cells and subsequent infection [43,44]. Additionally, the highly hydrated nature of mucins allows the trapping of microorganisms, facilitating their clearance from the epithelial surface [45]. Furthermore, the regulation of mucin secretion and composition is finely tuned to respond to various physiological and pathological stimuli. For example, in response to inflammation or infection, there is often an upregulation of mucin production to reinforce the protective barrier [46]. The dysregulation of mucin expression patterns, on the other hand, can lead to diseases such as cystic fibrosis, inflammatory bowel disease, and certain cancers [45,47,48].

### 2.1. General Features of Transmembrane Mucins

#### 2.1.1. Extracellular Domain

Transmembrane mucins are distinguished by their extracellular domains, which are rich in tandem repeat sequences composed predominantly of serine (Ser), threonine (Thr), and proline (Pro) residues [13,49]. These sequences are heavily O-glycosylated, which serves to shield the protein backbone from proteolytic degradation by bacteria and host proteases, thereby contributing to the formation of a robust barrier [50]. The glycan composition of mucins can vary significantly due to the differential expression of glycosyltransferases, resulting in a wide array of oligosaccharide structures. The terminal glycan residues, such as Gal, GlcNAc, Fuc, GalNAc, and sialic acid, can define blood group antigens and histo-blood group antigens, resulting in the A, B, and H as well as Lewis a, b, x, and y epitopes on mucins, within cells, and on erythrocytes [51,52]. This extensive process confers mucins with their protective and lubricative properties. In the upper gastrointestinal (GI) tract, mucins form a protective barrier against the harsh, acidic environment of the stomach, preventing damage to the epithelial cells [40]. In the lower GI tract, particularly the colon, mucins create a physical barrier that separates the epithelial cells from the gut microbiota, protecting the host from bacterial invasion and antigenic stimulation [41,42].

#### 2.1.2. SEA Domain

The extracellular domains of most transmembrane mucins, such as MUC1 and MUC16, include a sea urchin sperm protein, enterokinase, and agrin (SEA) domain [53]. This domain is located between the O-glycosylated tandem repeats and the transmembrane domain [54]. They undergo autoproteolytic cleavage in the endoplasmic reticulum [55], leading to the formation of an extracellular α-chain and a β-chain that remain noncovalently associated [56]. The SEA domain protects epithelial cells from mechanical stress by breaking before the apical membrane ruptures [56,57]. Additionally, it facilitates the release of the mucin extracellular domain, which is often detected in biological fluids such as serum and the lumen of the intestinal tract. This shedding process is frequently observed in metastatic carcinoma, inflammatory bowel disease, and cystic fibrosis, indicating a pathological relevance [58,59,60,61] (Table 1).

#### 2.1.3. EGF-like Domains

Many transmembrane mucins contain epidermal growth factor (EGF)-like domains, which are characterized by six cysteine residues that form three disulfide bonds [67,68]. Through interaction with EGF receptors and the activation of receptor signaling pathways, EGF-like domains are crucial for mucosal repair and mucin expression regulation [69]. For example, MUC4, which lacks a SEA domain, contains three predicted EGF domains that facilitate repair and regeneration processes in the gastrointestinal and respiratory tracts [70]. The release of the extracellular domain may enable these EGF domains to interact with their ligands more effectively, potentially playing a role in distant signaling like cytokines.

#### 2.1.4. Intracellular Domain

The intracellular domains, which are often referred to as the cytoplasmic tail of transmembrane mucins, are integral to cellular signaling pathways [20]. They contain PDZ-binding motifs that facilitate the trafficking and anchoring of receptor proteins, thereby organizing signaling complexes at cellular membranes [71,72]. In the gastrointestinal tract, the PDZ-binding motifs of MUC3, MUC12, and MUC17 compete with those of cystic fibrosis transmembrane conductance regulator (CFTR) chloride channels for adaptor proteins that regulate lysosomal degradation [73]. Furthermore, these cytoplasmic tails are subject to phosphorylation, mediating interactions with other proteins [20]. For instance, the phosphorylated cytoplasmic tail of MUC1 competes with E-cadherin for β-catenin binding, disrupting cell–cell adhesion and promoting cell detachment and growth [74]. Moreover, transmembrane mucins such as MUC1, MUC13, and MUC16 can localize to the nucleus, where their cytoplasmic tails influence transcription factors and regulatory proteins, contributing to cellular processes such as differentiation and proliferation [20] (Table 1).

#### 2.1.5. Glycosylation

**Figure 1:**
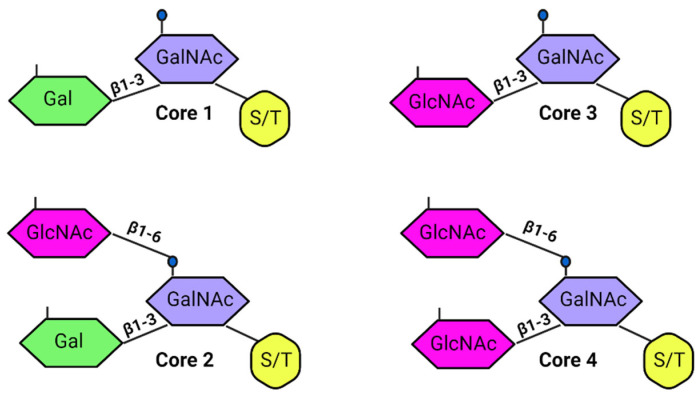
Four types of mucin core in human intestinal tract. The mucin core is composed of N-acetylgalactosamine (GalNAc) with different variations in galactose (Gal) and N-acetylglucosamine (GlcNAc) linkage via ß1-3 or ß1-6 [13].

In humans, eight core mucin structures have been identified, with cores 1 to 4 being most prevalent in the intestinal tract [13,75]. These core structures can be elongated with various combinations of Gal-GlcNAc residues, forming either ß1-3 (type 1 chain) or ß1-4 (type 2 chain linkages [50,51]. This elongation process leads to the formation of a protective glycan coat that shields the mucin protein core from enzymatic degradation and maintains its hydration and viscosity [76] (Figure 1).

The O-glycosylation process, which occurs in the Golgi apparatus, begins with the addition of N-acetylgalactosamine (GalNAc) to the hydroxyl groups of Ser and Thr residues. This is followed by the sequential addition of other sugars, including galactose (Gal), N-acetylglucosamine (GlcNAc), fucose (Fuc), and sialic acid [1,77,78]. Proline residues help stabilize the mucin polypeptide in an extended conformation, facilitating O-glycosylation. This process results in the formation of diverse glycan structures, which protect the protein core from proteolytic degradation, enhance solubility and hydration, and contribute to the gel-forming properties of mucins [76,79,80] (Table 1).

#### 2.1.6. Signaling Roles

**Figure 2:**
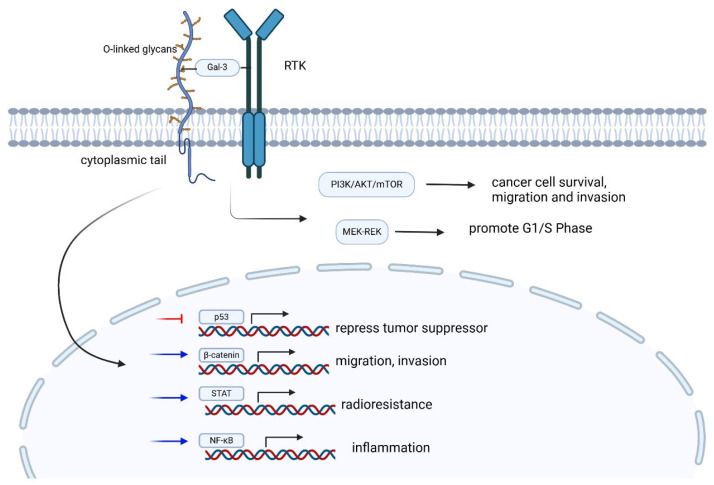
Downstream signaling of MUC1. The cytoplasmic tail of MUC1 interacts with various RTKs to activate PI3K/AKT/mTOR pathway, as well as MEK-REK pathways to promote cancer cell progression. The C-terminal of MUC1 also regulates the transcription of p53, β-catenin, STAT, and NF-kB to promote cancer cell migration and invasion. The red arrow indicates inhibition of transcription, while the blue arrow indicates promotion of transcription.

In addition to the protective functions, transmembrane mucins also play critical roles in transducing signaling pathways [81]. With numerous potential sites of phosphorylation and through various interactions with RTKs, such as hepatocyte growth factor receptor (Met), epidermal growth factor receptor (EGFR), and platelet-derived growth factor receptor β (PDGFR β), the cytoplasmic tail of MUC1 executes a wide array of phosphorylation signaling patterns [82,83,84]. Though the precise function of these phosphorylation remains unknown, it has been discovered that MUC1 has the potential to translocate to the nucleus and regulate the expression of transcription factors, such as p53, Wnt/β-catenin, the signal transducer and activator of transcription (STAT), and nuclear factor (NF)- *κ*B RelA pathways [85,86,87,88]. Through interaction with EGFR, ErbB2, and RTK at the cell membrane, MUC1 is involved in the activation of PI3K-AKT and MEK-ERK pathways [88,89,90,91] (Figure 2).

Though MUC4 lacks the SEA domain, it contains EGF-like domains with tyrosine and serine residues in its cytoplasmic tail that serve as potential sites of amino acid phosphorylation [92,93]. MUC4 was found to interact with ErbB family receptors and initiate signaling cascades involving RTK ErbB-2 (ErbB2/HER2) and ErbB3/HER3 [94,95], which leads to the cellular differentiation, proliferation, and inhibition of apoptosis. These MUC4-induced signaling pathways were found to protect tumor cells from HER2-targeted therapy in studies of cancer treatments [95,96]. In addition to HER2, MUC4 also interacts with HER3, which activates PI3K-ERK and focal adhesion kinase (FAK)-associated pathways, resulting in proliferation, metastasis, and angiogenesis in pancreatic cancer cells [94,97,98].

Similarly to MUC1, MUC16 is another transmembrane mucin with SEA domain, the largest core protein in size and longest mucin-type tandem repeats [99,100]. They also contain cytoplasmic tails that enable several potential phosphorylation sites [9]. MUC16 participates in signaling pathways at the cell surface and the nucleus via translocation [101]. It has been found that the cytoplasmic tail of MUC16 interacts with Janus kinase 2 (JAK2) and increases the activities of STAT3. These modulations induce cellular proliferation, the inhibition of apoptosis, and promote the metastasis of breast and pancreatic cancer cells [65,102]. MUC16 also interacts with Src and tyrosine protein kinases, which results in the phosphorylation of tyrosine in the MUC16 cytoplasmic tail, the shedding of the extracellular domain, and the dysregulation of β-catenin and E-cadherin [103,104].

#### 2.1.7. Cleavage and Shedding

In MUC1, the transmembrane domain is proteolytically cleaved after synthesis, creating an α and β subunit that binds in a noncovalent fashion [105]. This cleavage occurs at the SEA domain, which is vital for the proper maturation and function of mucins [106]. The EGF-like domain of MUC4 is also proteolytically cleaved in a manner essential for its complex formation [70,92,107]. The shedding of mucin extracellular domains can occur via proteolytic cleavage near the plasma membrane by sheddases or metalloproteases such as TNF-α converting enzyme/A Disintegrin and Metalloprotease-like (TACE/ADAM17) and membrane-type 1 matrix metalloproteases (MT1-MMP) [108]. These cleavages of the SEA domain, which can be triggered by mechanical force, microbial interactions, alterations in pH, ionic concentration, hydration, or inflammatory stimuli, also require serine, threonine, or cysteine at the proteolytic site [106]. As a result, cleaved mucin extracellular domains function as decoy receptors for pathogens at the mucosal surface, preventing pathogen adhesion to epithelial cells (Table 1).

### 2.2. Role of Mucins in Normal Physiology

Biophysically, the heavily glycosylated mucins contribute to the viscoelastic properties of the mucus gel [9]. In general, mucins lubricate, moisturize, and protect the surface of mucus from pathogens, prevent the rupture of the epithelium barrier, and remove invaders [13]. The heavily glycosylated mucins are commonly found in the gastrointestinal tract, lungs, salivary glands, sweat glands, and breast [3]. Typically, the structural pattern of *O*-glycosylation determines mucin classification and function. For example, secretory mucins, such as MUC2, are cysteine-rich and secreted by goblet cells in the airway and intestine. These cysteine-rich regions allow the formation of a complex covalent structure that protects the intestinal epithelium [109]. On the other hand, membrane-bound mucins, such as MUC1 and MUC3, are synthesized by epithelial cells and integrated into the apical plasma membrane. The C-terminal trans-membrane anchor on membrane-bound mucins is important for cellular signaling, allowing the coordination of cellular responses, such as cell proliferation, differentiation, apoptosis, or product secretion [110].

The outer mucin layer provides a physical barrier against microorganisms and irritants while maintaining the local microenvironment. For example, the sulfate-rich mucins are also more acidic and contribute to the protective function against mucin-degrading bacteria [111]. Furthermore, MUC5AC and MUC6 in the stomach protect its lining from gastric acid [112,113] by holding the secreted bicarbonate ion in their mucin layers [114]. By increasing mucin sialylation, the duodenal mucin prevents the diffusion of gastric acid [115] and maintains the optimal alkaline environment in the intestine.

In addition to the formation of physical barriers, mucins also release active molecules to initiate immune defense and mediate processes of inflammation and repair [116,117,118]. The release of trefoil factors (TFFs) increases mucus viscosity [119], promotes wound healing, and reconstitutes damaged mucus epithelium [120,121]. Besides restorations, the TFFs also inhibit apoptosis, promote cellular motility, and promote differentiation [122]. By secreting cells with IgG Fc binding proteins and IgA, mucin also plays a role in immune surveillance along the GI tract [123,124].

## 3. Altered Mucin Expression in Cancer

There are characteristic differences between healthy and cancerous tissues, especially in the localization, glycosylation, electrophoresis, and transcriptional expression profiles of mucin in the apical surface [4]. The overexpression of mucin provides oncogenic signals that favor the initiation of tumor progression [125]. For example, MUC1 stabilizes β-catenin and promotes nuclear translocation to active Wnt genes that drive the proliferation, invasion, and metastasis of intrahepatic cholangiocarcinoma [126]. It has been speculated that altered MUC2 expression, in combination with genetic factors, may increase colorectal cancer (CRC) risk by creating a microenvironment suitable for tumor growth and metastasis [127]. The decrease in expression of MUC5AC mucin is also correlated with a poorer prognosis of gastric cancer [128]. Alterations in MUC4 and MUC1 are noted in bladder cancer, pancreatic ductal adenocarcinoma, and oral squamous cell carcinoma [129,130,131,132]. Mucin can also cause abnormalities in the mucin network and disturb signaling pathways, leading to changes in the biomechanical properties of pancreatic tumors [133]. For instance, MUC1 sterically disrupts the PAR3-PAR-6-aPKC complex, resulting in the loss of tight junction and cellular polarity. As a result, mucins interact with growth factors and perpetuate oncogenic signaling [27,74]. In this section, we will delve into the role of mucin in cancer development.

### 3.1. Upregulated Mucins in Cancer

Upon the examination of the cancer cell membrane, several mucins are overexpressed and secreted to the cell surface [4,46]. In addition to the protective barrier, some membrane-spanning mucins also serve as cell surface receptors that aid in the survival, differentiation, and metastasis of cancer cells [4,95,134,135,136].

Current studies have found a profound association of MUC1 overexpression in numerous cancer types. In the study of mucin expression in mouse models, MUC1 overexpression was associated with the metastasis of pancreatic adenocarcinoma to the lung, liver, and peritoneal organs [137], as well as increased multidrug resistance in pancreatic cancer cells [138]. In mammary gland tumors, the upregulation of MUC1 also leads to tumorigenesis via its interaction with EGFR and the downstream activation of MAP kinase (MAPK) [83]. By phosphorylating the cytoplasmic tail of MUC1 at the YEKV motif, EGFR facilitates the binding of MUC1 to β-catenin, which enhances tumorigenesis [139]. In intrahepatic cholangiocarcinoma (ICC), the upregulation of myeloid leukemia factor 1 (MLF1) is significantly correlated with the upregulation of MUC1 expression involving the EMT of cancer progression [140]. The increased cell migration and evasion results in MUC1-induced EMT through the increased expression of vimentin and β-catenin [141]. In ovarian cancer, the upregulation of MUC16 activates the PI3K/Akt signaling pathway and promotes the expression of mesenchymal markers, such as N-cadherin, vimentin, and Snail, leading to increased proliferation, invasion, and metastasis [142]. By inducing DNA methyltransferase (DNMT) 1 and 3b, MUC1 also contributes to the suppression of tumor-suppressor genes via promoter-specific DNA methylation [143]. A high level of VEGF expression was also found in tumors with increased MUC1 via the AKT pathway [144]. As MUC1 is overexpressed, the phosphorylated Ser/Thy at the cytoplasmic portion of MUC1 conducts signals through ß-catenin and MAPK pathways [145]. The overexpression of MUC4 and MUC5AC at mRNA and protein levels was also observed during the progression of pancreatic intraepithelial neoplasia (PanIN) [146,147].

### 3.2. Downregulated Mucins in Cancer

Acting as a tumor suppressor, the MUC2 knockout mice models showed early development of GI tumor and invasion of carcinoma, along with a reduced number of goblet cells, decreased apoptosis, and the increased migration of intestinal epithelial cells [148]. In salivary gland tumors, reduced MUC-4 expression has been associated with higher-graded tumors [64]. Studies on the immunohistochemistry of colon cancer also revealed that the low expression of MUC2 is associated with colon tumor and less favorable disease outcome, and lack of expression of MUC5AC and MUC6 were associated with worsened stages of CRC [149]. It is worth noting that there were conflicting results regarding the prognostic values of MUC5AC in gastric cancer. Some studies revealed that a lower MUC5AC expression results in better survival, but some revealed that decreased MUC5AC expression results in poor prognosis [150,151]. In a meta-analysis performed by Zhang et al. in 2015, they concluded that decreased MUC5AC expression has a significant correlation with lymph node metastasis, aggressive histopathological patterns, and poor survival in gastric cancer patients [128].

## 4. Mucins in Cancer Metastatic Cascade

**Figure 3:**
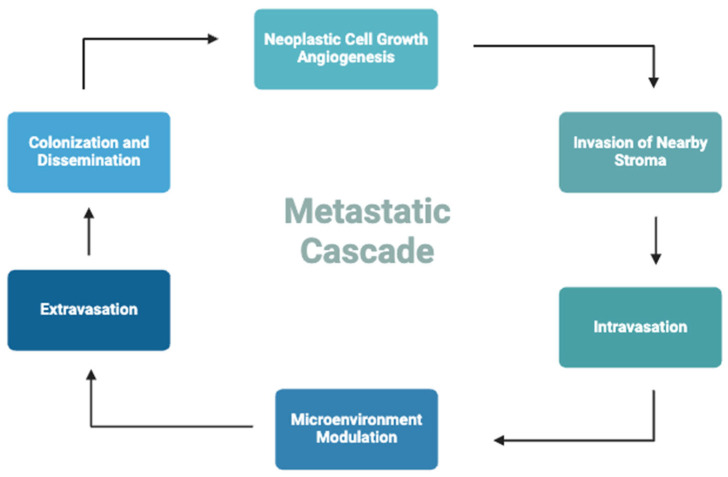
A schematic representation of the hematogenous metastatic cascade. Neoplastic cell growth and angiogenesis supply blood and nutrients that create an optimal tumor microenvironment for cancer cell growth, intravasation, migration, extravasation, and colonization at distal organs [158].

To date, mucin expressions have been well documented in many cancer types, where they are known to have key roles in host cell growth, differentiation, mobility, immune response, and apoptotic escape [4,95,134,135,136]. The role of mucin in cancer metastasis has been characterized in MUC1 and MUC16. MUC1 modulates the transcription of cell adhesion molecules and signaling pathways, promoting cancer cell migration, invasion, and immune evasion [87]. The cell surface-associated glycopeptides and extracellular signals derived from the proteolytic processing of MUC1 on the cancer cells alter the cytotoxicity and migration of natural killer (NK) cells and T-cells by modulating NK group 2D (NKG2D) ligands and β-catenin, respectively [74,152,153]. The downregulation of NKG2D ligands on tumor cells reduces NK cell recognition and cytotoxicity in hepatocellular carcinoma [154]. In colorectal and pancreatic cancer, the recruitment of regulatory T cells suppresses cytotoxic CD8+ T cells and promotes immune tolerance, providing an opportunity for uncontrolled cell growth and migration [155,156]. The role of MUC16 in metastasis involves regulating the expression levels of E-cadherin and vimentin [103,104], inhibiting the release of apoptotic ligands from breast cancer cells [102], promoting the nuclear translocation of JAK2 in pancreatic cancer cells [65], and modulating immune evasion [157]. Hallmarks of cancer metastasis include the growth of neoplastic cells, angiogenesis, intravasation into circulation, extravasation, and colonization at a secondary site. These series of processes are termed “metastatic cascade” [158] (Figure 3). In this section, the role of mucin glycoproteins in the progression and metastasis of cancer and their functions, as well as their therapeutic implications, will be discussed in depth [159] (Figure 3).

### 4.1. Cancer Cell Invasion and Migration

MUC4, MUC1, and MUC16 are involved in the regional invasion and migration of cancer cells through various signaling pathways. In the study of pancreatic cancer, the inhibition of MUC4 expression resulted in reduced tumor metastasis by enhancing cell proliferation and reducing apoptosis [134,160]. MUC1-C, the C-terminal domain of MUC1, functions as an oncoprotein, promoting the upregulation of genes and proteins involved in signaling pathways that are dysregulated in advanced cancers. In studies of mammary tumors, MUC1 augments the dimerization of Her 2 and EGFR, which has been associated with invasive papillary mucinous neoplasm [161,162]. Additionally, MUC16’s interaction with JAK2, Src, and FAK was found to facilitate tumor growth and metastasis [102,103,163]. Lakshmanan et al. also identified that MUC16 enhanced the endothelial cell and p-selectin binding of pancreatic cancer cells to favor the metastatic spread of pancreatic ductal adenocarcinoma [164].

### 4.2. Cancer Cell Adhesion and Colonization

In the study of metastatic breast cancer, the under-glycosylated MUC1 expresses sialyl Lewis x and a, which slows extravascular rolling and enhances the adhesion to distant sites [165]. Galectin-3 (Gal-3), an endogenous lectin, helps stabilize MUC4 mRNA in the cytoplasm, which is highly expressed in pancreatic cancer cells [166]. In addition to MUC4, Gal-3 is also a ligand for MUC1 in epithelial cells, which induces MUC1 cell surface polarization, increased cancer cell aggregation, and adhesion to vascular endothelium [167,168,169].

### 4.3. Immune Evasion and Tumor Microenvironment

The phenomenon of immune modulation and evasion by cancer cells is fundamental to cancer development. Tumors exploit the immune cells’ ability to suppress responses through an adaptive process that is known as immunoediting [170]. By remaining dormant under immunosurveillance, coexisting with the immune system, bypassing checkpoints, and escaping from immune attacks, tumors began to proliferate and migrate in an unregulated fashion [170]. In the context of mucin, the expression of sialosyl-Tn (STn) antigen expressed by mucin significantly inhibits the cytotoxicity of natural killer (NK) and lymphocytic cells [171,172]. Additionally, MUC1 disrupts the interaction between tumor cell antigen and MHC-I receptors in innate immune cells. Besides MUC1, MUC16 also reduces T effector cell function by binding to toll-like receptors on dendritic cells [173].

The tumor microenvironment (TME) is composed of non-cancerous cellular and structural components, including cancer-associated fibroblasts (CAFs), adipocytes, vascular endothelial cells, and immune cells, along with the extracellular matrix (ECM) and extracellular vesicles these cells secrete. This three-dimensional scaffold not only supports cancer cell survival and proliferation but also plays a pivotal role in the transition from normal to malignant tissue [174,175]. Within the TME, mucins serve as critical ligands and signaling molecules for immune cells. They effectively modulate mechanisms of immune surveillance and immune escape. For example, MUC1 modulates TME through its interaction with lectin Siglec-9 on myeloid cells, inducing monocytes to secrete Il6, macrophage colony-stimulating factors (M-CSF), and plasminogen activator inhibitor (PAI-1) [58]. The altered expression of mucins in various cancers impacts their utility as potential biomarkers for early diagnosis and therapeutic monitoring [173,176]. These therapies include immune checkpoint inhibitors and chimeric antigen receptor (CAR) T-cell therapies, which are at the forefront of personalized cancer treatment strategies. The intricate nature of the TME can influence the effectiveness of therapeutic interventions, including the viability of surgical resection, which remains the primary curative treatment in many cases.

### 4.4. Mucins and Angiogenesis

By forming new blood vessels, angiogenesis provides a route for nutrient delivery, waste removal, and immune surveillance [177]. To support the growth and enhance the invasive potential of tumors, malignant cells overexpressed angiogenic factors, such as VEGF and their receptors (VEGFRs), to promote the neovascularization and remodeling of tumor blood vessels [178]. As many cancer cells thrive in a hypoxic environment, the increase in MUC1 expression helps induce the increase in VEGF, connective tissue growth factor (CTGF), and platelet-derived growth factor (PDGF-β) to support tumor angiogenesis [179]. More specifically, the overexpression of MUC1 in pancreatic and breast cancer cells interacts with signaling pathways, such as the MAPK, JAK/STAT, and the PI3K/AKT/mTOR pathways, and VEGF co-receptor, neuropilin-1, to promote the generation of endothelial cell and ectopic blood vessels [144,180]. Hypoxia-inducible factor-1 alpha (HIF-1*α*) is a transcription factor that is normally deactivated in an oxygen-rich environment but activated in an oxygen-poor environment and upregulated gene expression related to cancer progression, such as angiogenesis and metabolism reprogramming [181]. The upregulation of MUC1 expression is one of the downstream effects of HIF-1*α* activation, which is consistent with MUC-1-induced cancer cell migration and invasion [182].

## 5. Mucins in Different Types of Cancer

In normal physiological contexts, mucin’s long glycan chains create a water-rich antiadhesive mucus that protects tissues from dehydration, infections, and physical damage [37,46]. However, the deregulation of mucin glycosylation and altered mucin cellular localization are observed in numerous cancer types [129,130,131,132]. Cancer-associated mucins, especially those expressed at the cell surface, imped the cellular clearance of low-sialylated cellular proteins, deregulate the expression of immune activation markers, induce the secretion of VEGF, CTGF, metastatic markers, and inhibit cytotoxic lymphocytes [171,172,178,179]. The current chapter shows the importance of various mucins (MUC1, MUC3A, MUC4, MUC4β, MUC5AC, MUC5B, MUC7, and MUC16) and their mucin-bound carrier proteins in different cancers (Table 2).

### 5.1. Breast Cancer

In normal breast epithelial cells, MUC1 is expressed at the apical surface at low levels [183], in which their high molecular weight glycoproteins with complex O-glycans function as cytoprotective and lubricating agents [20]. In studies of breast cancer cells, the increased activity in sialyltransferase (STGal-I) was observed in the exposure of otherwise cryptic peptide epitopes expressed by MUC1 [184]. Moreover, the mucin expressed by breast cancer cell lines has a shorter sidechain that consists of Galβ1-3f, which is also associated with the addition of sialic acid by STGal-I that terminates the glycan chain extension [185]. Among the various N-acetylglucosaminyltransferases (GalNAcTs), GalNAcT6 has been identified to stabilize MUC1 [186], and GalNAcT14 is highly expressed in breast cancers [181,187]. The overexpression of ST3Gal-I also leads to the increased expression of sialyate core 1 in mucins and is associated with the tumorigenesis of breast cancer [188]. MUC1-ST, a tumor glycoform of MUC1, interacts with siglec-9 expressed by tumor-associated macrophages [189], which activates MEK-ERK and contributes to the poorer prognosis of breast cancer [58]. With the extensive involvement of MUC1 in breast cancer, recent studies have focused on the role of mucin in immunotherapy [190], which will be further discussed in a later chapter (Table 2).

### 5.2. Colorectal Cancer

In normal colon mucosa, mucins are equipped with Core 1-4 of O-glycans [191]. These 4 O-glycans can be synthesized and modified by different families of glycosyltransferases [192]. The increased expression of Tn and STn antigens correlate with the progression of colonic malignancy and are used as markers for poorly differentiated mucinous carcinomas [193,194]. More specifically, the expression of the sialyl Lewis x epitope in colon cancer is associated with poor survival [195]. Similarly to MUC1 discussed in breast cancer, MUC16 has also been identified to bind to Siglec-9 [196], attenuating T and NK cell functions to help the survival of cancer cells.

Aside from the alteration in the mucin glycosylation, the downregulation of MUC2 is also associated with metastasis and poor prognosis in colon cancer [197,198]. With abundant and variable O-glycan, MUC2 is a major mucin in the colon mucus that serves as an essential protective physical barrier against bacteria and inflammation [199]. The suppression of MUC2 expression is found to be associated with IL-6 overexpression, which leads to inflammation and tumor growth [200]. In addition to MUC2, the abnormal expression of MUC5AC is also observed in colorectal cancer [201]. In histological studies of the colorectal adenocarcinoma differentiation, the MUC2 level was found to decrease, and MUC5AC tends to increase when CRC progresses from moderately to poorly differentiated adenocarcinoma [202]. The overexpression of MUC5AC is associated with BRAF mutation [203] and the activation of the CD44/β-catenin/p53/p21 signaling pathway that contributes to tumorigenesis and chemoresistance [204]. The increased MUC5 and decreased MUC2 expression in CRC is also associated with the increased prevalence of lymph node metastasis and advanced tumor stages [201] (Table 2).

### 5.3. Pancreatic Cancer

Pancreatic cancer (PC) is characterized by a decline in 5-year survival rate and ranks as the seventh leading cause of death among all cancers in America [205]. In the search for biomarkers, current research has illustrated the significance of glycoproteins, such as CA19-9 and CA125, in PC diagnosis and prognosis markers [205]. Produced as an aberrant version of disialyl Lewis a sialic acid residual, CA19-9 has been studied extensively as a promising tumor marker for PC diagnosis and staging [206,207]. CA125 is another high molecular weight glycoprotein that is part of the MUC16 extracellular domain, of which studies of anti-MUC16 monoclonal antibodies have shown to reduce the actives of pancreatic tumors [208]. Though the mechanism remained unknown, a meta-analysis by Huang et al. has identified a significant association between high MUC4 expression and poor prognosis of metastatic pancreatic cancer [209]. In studies of PC in mice models, MUC4 knockout cells had less tumorigenicity and were more sensitive to gemcitabine treatment [63].

Though expressed in the apical membrane of the intralobular duct, glycoforms of MUC1 are undetectable in the main pancreatic duct [210]. However, in studies of pancreatic cancer, high MUC1 expression has been strongly associated with the invasiveness of pancreatic cancer [211,212]. The cytoplasmic tail of MUC1 is known to interact with β-catenin and EGFR to potentiate cell proliferation, motility, and chemoresistance in subtypes of PC via AKT and BCL-2 pathways [213,214]. Aside from cancer progression, MUC1 is also involved in chemoresistance in PC cells through the inhibition of BRCA1 to enhance glucose utilization [211] (Table 2).

**Table 2:** Summary of mucin deregulation in cancer.

Cancer	Mucin and Mucin-Associated Deregulation	Proposed Mechanisms
Breast	MUC1: GalNAcT14 [187], GALNT6 [186] MEK/ERK [58], MAPK, JAK/STAT, and PI3K/AKT/mTOR pathways [144,180], neuropilin-1 [187,215] MUC16: Sialyl Lewis x and [165], and JAK2 [65,102]	Stabilization of MUC1 [186,187]. Increase tumorigenesis [58,187], inhibit apoptosis [65], angiogenesis [215]. Enhance cellular adhesion for distal metastasis [165]. Increase G2/M transition for cellular proliferation [65].
Colorectal	MUC2: IL-6 overexpression [201] MUC5AC: CD44/β-catenin/p53/p21 signaling pathway [201,202] MUC16 [196]: Sialyl Lewis x epitope [195]	Inflammation and tumor growth [200] Tumorigenesis and chemoresistance [204] Invasion and metastasis [195], attenuation of NK and T cell [196]
Pancreatic	MUC1: β-catenin and EGFR, AKT and BCL-2, BRCA1, MAPK, JAK/STAT and the PI3K/AKT/mTOR pathways, and neuropilin-1 [210,213,214] MUC4: integrin-mediated cell adhesion [134], HER2/neu [160], MUC16: PI3K/AKT/mTOR pathways [180], Treg [216]	Enhance cell proliferation, motility, chemoresistance, glucose utilization, and angiogenesis. [210,213,214] Inhibit integrin-mediated cell adhesion [134], increased cellular proliferation and metastasis [160]. Increase tumor survival [180], promote immunosuppressive tumor microenvironment [216]

## 6. Experimental Models and Techniques in Mucin Research

To investigate the role of mucin in cancer development, cell culture models are commonly used to study mucin expression, glycosylation, and secretion in cancer progression, metastasis and treatment resistance. Some commonly used adenocarcinoma cell lines include those coming from gastrointestinal, pancreatic, breast, and lung cancers [136,217,218,219]. For example, CAPAN-1 and CAPAN-2 cell lines, derived from pancreatic ductal adenocarcinoma, expressed high levels of MUC1 and MUC4, which were used in studies of chemotherapist-resistant pancreatic cancer [218]. In addition to traditional 2D cell models, recent studies have investigated the use of 3D multicellular spheroid cell models in cancer research [220]. With the ability to develop metabolic gradients in spheroid models, this technique enabled researchers to mimic in vitro environments under in vivo conditions, which could also resemble avascular stages of solid tumors [221,222,223]. Three-dimensional spheroids with a combination of siRNA transfections, lentiviral transductions, and quantitative PCR are valuable tools in gene functional and mechanistic assays. Taking this into account, spheroids can serve as a unique form of in vitro models to improve the relevance of in vitro research for human disease in an immunocompetent and controlled condition [39,114,117].

## 7. Mucins as Cancer Biomarkers

Through genetic, epigenetic, proteomic, glycomic, and microbial changes, the expression of mucin is altered in different disease stages [224,225,226]. By understanding the complex interplay of protein structure and mucin function in different pathophysiological conditions, such as colorectal cancer, we hope to elude markers that enable the early diagnosis of serious illness [125].

### 7.1. Current Mucin Biomarker

The search for more specific markers for the early detection of tumorigenesis has led to an interest in altered mucin protein glycosylation. The developments of novel detection methods for mutated O-glycan epitopes and the currently available commercial assays enable the assessment of mucin-based glyco-markers for cancer diagnosis, prognosis, and therapy monitoring [227]. For instance, MUC1, which is overexpressed in the colon, breast, lung, pancreatic, bladder, ovarian, bladder, gastric, and esophageal cancer [228,229,230,231,232,233,234], has been reported as a critical protein for the development of pluripotency, platform proteins secreted by tumor-associated surface proteases [58,235]. This evidence supports that MUC1 can be considered as an early diagnostic biomarker (lung adenocarcinoma [236], pancreatic [237,238], or ovarian [239] cancer) or as a prognostic biomarker (bladder carcinoma [240], node-negative breast [241], lung squamous cell [242], and esophageal cancer [243].

Numerous studies have shown that patterns of mucin expression may have a role in tracking pathologies or phenotyping when a detailed histological tumor definition is not pervasive. For example, recent evidence has suggested that mucin expression may be indispensable in cancer biology as a diagnostic marker for genitourinary squamous cell carcinomas and adenocarcinomas [240,244]. More specifically, endocervical and endometrial mucin markers demonstrate widely varying and potential diagnostic accuracy [245,246]. In fact, by adjusting immunophenotypes, all adenocarcinomas (i.e., carcinomas with mucus-producing epithelia) of the uteri are split into two groups: papillary endocervical and endometrial adenocarcinomas (EMCA) [247]. Regarding ovarian carcinoma and cysts, MSA (UMA1) and MUC16 (CA125) can present equivocal test scenarios [248,249]. Tests are often negative in invasive serous carcinomas for ovarian cysts, endometrioid cysts, and borderline lesions [250]. The usual differences in disease biology are confusing players in cancer diagnostics and cancer cell propagation.

The two carbohydrate antigens (CAs), MUC5AC and carcinoembryonic antigen (CEA), are more frequently detected in esophageal than in gastric cancers, according to both immunohistochemical and biochemical data [251]. In most cases, gastric MUC5AC is reduced or absent [128], while CEA usually demonstrates a higher positivity in esophageal cancer than in gastric cancer [252,253]. The prognostic significance of these mucins is also partially different in the two tumor types, with MUC5AC being more helpful in gastric [128] and CEA in esophageal cancers [254].

### 7.2. Limitation of Mucin as Biomarker

Though the altered expression of mucin is detected in various cancer cells, there are several limitations of mucin as biomarkers for cancer diagnosis. Mucin expression varies across cancer types. The increased expression of MUC5AC in pancreatic and lung cancer is correlated with poor prognosis [255,256,257], while in gastric carcinoma, decreased MUC5AC expression correlates with poor prognosis [258]. The variability in the O-glycosylation profile in mucin post-translational modification and the lack of tissue specificity also pose challenges in utilizing mucin proteins as cancer biomarkers [259]. The lack of sensitivity in early cancer detection is another notable limitation. For example, while CA125, a MUC16 marker, has been widely used as a serum biomarker for ovarian cancer, the limited sensitivity makes it difficult to screen early-stage ovarian cancer [260,261]. While the detection of mucin protein backbone or O-glycans may yield low sensitivity and specificity, some studies have combined mucin and its O-glycan feature to increase specificity as cancer biomarkers [259]. Though mucin shows promise as a cancer biomarker, the variable expression across cancer types, limited specificity, and sensitivity make it challenging to definite diagnostic tools.

## 8. Therapeutic Targeting of Mucins in Cancer

The aberrant glycosylation of mucin, including increased N-glycan branching, sialic acid glycans, and shortened O-glycans, is the core signature of mucin-associated cancer cells [262]. Though the exact mechanisms are under investigation, this abnormality has been associated with proliferation [263], the loss of adhesion [264], and the evasion of immune surveillance [62] in cancer cells. With its unique patterns and cancer-associated epitopes, MUC1 has been studied as a potential therapeutic target for mucin-associated cancer cells [265].

### 8.1. Current Strategies and Challenges

Widely expressed in many cancer types, MUC1 is heavily studied in cancer biology research. With the interaction between the MUC1 C-terminal subunit (MUC1-C) and receptor tyrosine kinases at the cell membrane, the MUC1-C inhibitor has been studied as a potential therapeutic intervention for breast cancer [88]. Recent studies have examined the use of monoclonal antibodies (mAbs) and anti-mucin vaccines in treating cancers with overexpressed mucin proteins. For example, AS1402, an IgG mAbs that binds to aberrant extracellular MUC1 peptide that is not normally exposed in normal breast, has demonstrated its efficacy in Phase I and II clinical trials [266,267]. In treating pancreatic cancer, mAb and TAB004, which target tumors associated with MUC1 (tMUC1), have shown a reduction in colony formation in the PDAC cell line [268]. In addition to mAbs, vaccines against cancer have also been developed. Examples of MUC1-based cancer vaccines include subunit vaccines, DNA vaccines, viral vector vaccines, dendritic cell vaccines, and glycoprotein vaccines [269]. Julien et al. also observed a significant delay in tumor growth when treating tumor cells with a coupled sialyl-Tn vaccine [270]. Preclinical studies on adjuvanted MUC1 subunit vaccines have shown their antitumor activities by inducing Th1 and NK cell response to suppress tumor cells in mice models [271,272,273]. Several subunit vaccines, such as L-BLP25, have undergone trials and shown improved survival outcomes in patients with non-small cell lung cancer and prostate cancer [274,275].

Current challenges in mucin-target cancer therapy include the immunosuppressive tumor microenvironment, the negative charge of the mucin barrier, and non-specific drug distribution systems. The immunosuppressive microenvironment has posed a challenge in the efficient delivery of MUC-1-based vaccines, which activate immune cells and exert cytotoxic pathways [276]. The negatively charged mucin also creates a diffusion barrier for positively charged drugs, leading to chemoresistance in cancer cells [277,278,279]. By disrupting mucin synthesis through the glucosaminyl transferase 3 inhibitor (GCNT3), it is hoped to improve drug delivery and delay PanIN progression [279]. However, its therapeutic potential requires further investigation. To overcome challenges in tumor penetration of antitumor mAbs, radiopharmaceuticals act as carriers to improve the stability and immune reactivity of antibody treatments [280,281]. Other shortcomings of cancer therapy include high toxicity and systemic side effects due to non-specific drug distribution. To overcome these challenges, recent studies have shown an improvement in the sensitivity of chemotherapy drugs by coupling mAbs with nanoparticles and chemokine receptor CXR4 antagonists [282].

### 8.2. Novel Approaches and Future Directions

With protein glycosylation playing a role in malignant transformation and immune tolerance, current studies have explored the therapeutic potential of chimeric antigen receptor T cells (CAR-T) in solid tumors [283,284,285]. Posey et al. demonstrated target-specific cytotoxicity activity of anit-Tn-MUC1 CAR-T cells in pancreatic xenograft models [283]. By transducing CAR-T cells with TAB004, Zhou et al. demonstrated a novel therapeutic approach to target tMUC1 in breast cancer by inducing the production of cytokines and chemokines to suppress tMUC1-positive tumors without disrupting normal breast epithelial cells [286]. The Tn-MUC1 targeted CAR-T cells also showed effectiveness in targeting and eliminating Tn-MUC1-positive intrahepatic cholangiocarcinoma in both in vivo and in vitro models [287]. Similarly to the mAbs and vaccine therapy mentioned above, the tumor-associated macrophages (TAM) in the tumor microenvironment inhibit the efficacy of CAR-T cells [288]. To target the TAMs and increase phagocytosis of tumor cells, CAR-macrophages (CAR-M) have been studied to enhance tumor antigen presentation and T-cell activation [289,290]. By adjuvating CAR-T and CAR-M therapy, it is hoped to improve the efficiency of cancer immunotherapy. Though in vitro models have shown promising results in the novel antitumor activities in targeting mucin-associated cancer cells, further investigation and clinical trials are warranted to evaluate the safety and efficacy of cancer treatments.

## 9. Conclusions and Future Perspectives

The complexity and versatility of mucin function and signaling pathways are essential for biological survival. Extensive glycosylation and glycopeptides mucins serve an essential role as a protective barrier and cellular signaling. Disruption in mucin expression, such as the upregulation, downregulation, and exposure of normally hidden mucin antigen on cell surfaces, leads to the malignant transformation of epithelial cells in breast, pancreatic, and colon cancer. While MUC1, MUC4, MUC5, and MUC16 are all involved in cancer progression, MUC1 has been extensively studied in its ability to modulate the innate immune system, develop tumor microenvironment, and promote tumor angiogenesis that contributes to the proliferation, invasion, and metastasis of cancer cells. While most mucins are upregulated in their association with cancer, the downregulation of mucin, such as MUC2, expression is also involved in pro-inflammatory behavior that favors cancer progression.

With mucin’s extensive involvement in cancer formation, many studies have investigated mucin’s potential to serve as a biomarker. Comparing mucin’s pattern of expression, immunophenotypes, histological presentation, and serum antigen levels are potential diagnostic and disease monitoring tools for clinicians. However, the variability in mucin glycosylation and lack of specificity in mucin expression pose challenges in early cancer detection. Despite current challenges in searching for highly sensitive and specific diagnostic mucin markers, recent discoveries and investigations into mucin-targeted immunotherapy through autoimmune antibodies and vaccines have yielded new strategies to improve diagnostics and therapeutics in mucin-associated cancers.

The involvement of mucin in cancer is extremely intricate, especially with its diverse glycosylation and cleavage patterns and involvement with numerous signaling pathways. The irregular dysregulation of mucin and the overlapping expression of mucin across different cancer types have made it challenging to characterize their diagnostic and therapeutic potential. Yet, recent discoveries in mucin’s role as biomarkers and therapeutic targets inspire further innovative approaches to cancer research. To encompass our current understanding of mucins, our study provided a comprehensive review of mucins and their association with cancer. Through a thorough discussion of the biochemical structure of mucin, we highlighted its significance in cellular communication within physiological, pathological, and oncogenic pathways. This review emphasized the role of mucin, especially MUC1, in modulating the tumor microenvironment and the immune system in favor of cancer proliferation. By discussing novel therapeutic approaches that target mucin-associated cancer and illustrating the current barriers in diagnostic and therapeutic modalities, we provide novel insights into the possible future directions of mucin cancer research.

## Figures and Tables

**Table 1:** The general features of transmembrane mucins.

Feature	Description	Mucin Examples
Structural Composition	Extracellular Domain: Rich in serine, threonine, and proline residues, heavily O-glycosylated. Forms a protective barrier against proteolytic enzymes, binds water, and forms gels. SEA Domain: Undergoes autoproteolytic cleavage, resulting in an extracellular α-chain and a transmembrane/intracellular β-chain.	Extracellular Domain (membrane-bound): MUC 1, 4, 16 [62,63,64,65] Extracellular Domain (secreted): MUC 2, 6, 19 [1,9]
		SEA Domain: MUC 1, 12, 13, [1]
		Intracellular Domain: MUC 1, 3, 12, 17, [20]
		ECF-like Domain: MUC 4, 12, 13, 17, [1]
Glycosylation	Extensively glycosylated to shield the protein backbone and maintain hydration. Glycosylation patterns vary, leading to different functional properties.	MUC 1, 4, 16 [1]
Signaling Roles	Intracellular tails participate in signaling pathways, influencing cellular responses. For example, the MUC1 tail can be phosphorylated, affecting cell adhesion and proliferation.	MUC 1 and 4 [66]
Shedding and Cleavage	Extracellular domains can be shed from the cell surface, especially during inflammatory responses or in cancer. Shedding is mediated by proteases and modulates signaling and interactions.	MUC 1, 4, 16 [20]
